# Looking for the Self in Pathological Unconsciousness

**DOI:** 10.3389/fnhum.2013.00538

**Published:** 2013-09-03

**Authors:** Athena Demertzi, Audrey Vanhaudenhuyse, Serge Brédart, Lizette Heine, Carol di Perri, Steven Laureys

**Affiliations:** ^1^Coma Science Group, Cyclotron Research Center and Neurology Department, University of Liège, Liège, Belgium; ^2^Department of Psychology, Behavior, and Cognition, University of Liège, Liège, Belgium; ^3^Department of Neuroradiology, National Neurological Institute C. Mondino, Pavia, Italy

**Keywords:** consciousness, self, neuroimaging, disorders of consciousness, default mode network, external awareness

## Abstract

There is an intimate relationship between consciousness and the notion of self. By studying patients with disorders of consciousness, we are offered with a unique lesion approach to tackle the neural correlates of self in the absence of subjective reports. Studies employing neuroimaging techniques point to the critical involvement of midline anterior and posterior cortices in response to the passive presentation of self-referential stimuli, such as the patient’s own name and own face. Also, resting state studies show that these midline regions are severely impaired as a function of the level of consciousness. Theoretical frameworks combining all this progress surpass the functional localization of self-related cognition and suggest a dynamic system-level approach to the phenomenological complexity of subjectivity. Importantly for non-communicating patients suffering from disorders of consciousness, the clinical translation of these technologies will allow medical professionals and families to better comprehend these disorders and plan efficient medical management for these patients.

## (Self) Consciousness in Non-Communicating Conditions

The scientific study of consciousness dictates that there is an intimate relationship between the mind and the brain (Feinberg, 2000; John, 2002; Freeman, 2007; Tononi and Laureys, 2009; Fingelkurts et al., 2013). Nevertheless, besides several attempts to define it, consciousness remains a difficult term to describe and different people may think differently about it (Demertzi et al., 2009). Here, we will define consciousness in an operational manner, namely consciousness is what is lost during dreamless sleep (Tononi, 2004). As such, consciousness is a matter of both waking states and experience, so that the less awake we get the less aware we become of our surroundings and ourselves.

Based on this definition, patients in coma are not conscious because they cannot be awakened. The linear relationship between wakefulness and awareness is violated in cases of severely brain-damaged patients who are in a vegetative state (VS) and minimally conscious state (MCS). Indeed, patients is VS, also coined as unresponsive wakefulness syndrome (UWS; Laureys et al., 2010), maintain awaking periods as evidenced by eye-opening and they will never respond to any visual, somatosensory, or auditory stimulation indicative of preserved awareness (Jennett and Plum, 1972). On the other hand, patients in MCS show fluctuating signs of awareness and non-reflex behaviors, such as visual pursuit and command following (Giacino et al., 2002). Importantly, in both clinical conditions patients remain unable to communicate with their environment in a functional manner. In the absence of subjective reports, how can one know whether patients in VS/UWS and MCS experience something and what these experiences are? In other words, can one claim that these patients retain a type of “core consciousness,” which provides them with a sense of self about here and now? (Damasio and Meyer, 2009). We think that the study of patients with disorders of consciousness offers a unique lesion approach to tackle the necessary neural correlates of self-consciousness. Our rationale lies on the argument that since clinical diagnosis shows that patients hold no subjective experience, the absence of subjective identity will be eventually reflected in patients’ brain function. As these patients are not able to communicate or show high-level cognitive function, we will here refer to self-consciousness as to its basic expression. In other words, as self-detection, namely when an organism can respond to stimuli with which is directly implicated or modify its behavior in ways which imply awareness of its own actions (Zeman, 2001). Accordingly, the employed experimental paradigms refer to the administration of self-referential stimuli (patients’ own name and own face) and the subsequent measure of brain responses to these stimuli with neuroimaging and electrophysiological techniques. The excellent spatial resolution which is offered by functional magnetic resonance imaging (fMRI) and positron emission tomography (PET), permits to better “localize” self-referential brain activity. Therefore, we will here focus on studies employing these neuroimaging methods to study residual self-consciousness in patients with disorders of consciousness. To date, such functional neuroimaging studies point to the critical recruitment of anterior and posterior midline cerebral areas in experimental paradigms employing self-referential stimuli. Activation of these midline regions is further observed during resting state conditions in healthy volunteers. This has led to the suggestion of a link between resting state activity and unconstraint self-related mentation. We will review these studies in patients and healthy controls, discuss the involvement of midline areas to the notion of self in patients and will propose that self-related cognition might be a matter of a system-level dynamic activity rather than activation of specific brain areas.

## Assessing Self-Consciousness in Non-Communicating Patients

Clinicians are offered with various clinical scales to detect sings of awareness at the bedside (Majerus et al., 2005). The Coma Recovery Scale-Revised (Giacino et al., 2004) is one of the most sensitive tool to diagnose and differentiate between patients in VS/UWS and MCS because it assesses all the defining criteria for MCS, such as visual pursuit (Seel et al., 2010). Nonetheless, it is not only a certain behavior that needs to be detected, but the way this is assessed seems to be equally important. For example, when visual pursuit was tested by means of a moving object, a moving person, and a moving mirror, more patients tracked their image in the mirror compared the other two stimuli and were hence considered as in a MCS (Vanhaudenhuyse et al., 2008). Similarly, to score sound localization with the Coma Recovery Scale-Revised, patients need to orient their head or eyes toward the source of the sound. When the patients’ own names were used, more oriented their head or eyes toward the examiner compared to the meaningless sound of a ringing bell (Cheng et al., 2013). These studies imply that self-referential stimuli are more effective to explore patients’ responsiveness and can influence the diagnostic process (also, see Laureys et al., 2007). To what degree, however, can one claim that these paradigms also reflect the, indirect, assessment of residual self-consciousness in this non-communicating clinical population?

One way to approach the answer is to measure patients’ brain responses and activation during sensitive experimental manipulations and compare them with that of healthy controls. If the cerebral pattern is indistinguishable between the two groups, then one has good reasons to believe that the extracted statistical maps reflect the same construct (Owen, 2013). Naturally, there are emerging legitimate concerns about the degree of confidence one can have on functional neuroimaging results, especially in the absence of subjective reports (e.g., Fins and Schiff, 2010). In addition, our limited understanding of the dynamic neural complexity underlying consciousness and its resistance to quantification in the absence of communication (Seth et al., 2008) makes it difficult to establish strong claims about self-consciousness in non-communicating patients. Nevertheless, the use of these technologies have shed light on the gray zones between the different clinical entities of consciousness and have revealed that not all patients can be considered unresponsive (Laureys and Boly, 2008; Gantner et al., 2012). For example, fMRI has been used to assist the diagnosis of patients with disorders of consciousness (Coleman et al., 2009), to detect preserved awareness in behaviorally unresponsive patients (Owen et al., 2006), and even to communicate with them (Monti et al., 2010).

Due to the difficulty to control voluntary eye-opening of patients, most neuroimaging studies employing self-referential stimuli restrict to the auditory modality (Table 1). In a PET study with one patient in MCS, the patient’s own name was presented next to baby cries and meaningless noise (Laureys et al., 2004). Passive listening to the own name recruited the activation of midline areas, such as precuneus and anterior cingulate/mesiofrontal cortex next to lateral parietal areas including language-related regions, such as Broca’s and Wernicke’s. Another *n* = 1 study with a patient in VS/UWS utilizing fMRI also showed that passive listening to the own name compared to other names, encompassed the activation of the medial prefrontal cortex bilaterally in parallel to temporo-parietal and superior frontal cortices (Staffen et al., 2006). Including more patients (*n* = 11), it was shown that all four patients in MCS and six patients in VS/UWS showed cerebral responses to their own names either in the anterior cingulate cortex (ACC) or in the caudal part of the ACC or the supplementary motor area (predefined regions based on brain responses of healthy controls) (Qin et al., 2010). Interestingly, those two patients in VS/UWS who exhibited activity in the caudal ACC evolved to a MCS at a 3-month follow up. Similarly, two patients in VS/UWS when listening to their own name showed cerebral activation extending to associative auditory cortex and also recovered to MCS (Di et al., 2007). Such brain activations, however, are atypical of the VS/UWS. Indeed, it has been shown that auditory processing of simple stimuli in VS/UWS refers to the activation of only auditory primary cortices whereas hierarchically higher-order multi-modal association areas are not activated (Laureys et al., 2000; Boly et al., 2004). Although caution should be paid on the accurate behavioral evaluation of these patients with standardized tools, like the Coma Recovery Scale-Revised (Table 1), there are cases of unresponsive patients where functional neuroimaging can precede the clinic (e.g., Owen et al., 2006). Taken together, these studies suggest that when activity of the anterior midline areas is recruited using the own name paradigm, this can work as prognostic marker (for a review, see Di et al., 2008).

**Table 1:** Studies showing brain responses to the presentation of self-referential stimuli in patients in vegetative state/unresponsive wakefulness syndrome (UWS) and minimally conscious state (MCS) by means of positron emission tomography (PET) and functional magnetic resonance imaging (fMRI) techniques (*indicates prognostic value).

Technique	Patients	Coma recovery scale-revised assessment?	Experimental contrast	Implicated brain regions	Reference
fMRI	4 MCS, 7 UWS	Yes	Passive listening to own name by familiar voice	In predefined regions of ACC, cACC, and SMA:	Qin et al. (2010)
				• In all 4 MCS and six UWS: signal changes in at least one the three regions • In 2 UWS: activity in cACC (clinical improvement to MCS at three-month follow up)*	
fMRI	4 MCS, 7 UWS	Yes	Passive listening to own name by familiar voice vs. baseline (machine noise)	• In all 4 MCS: primary auditory cortex extending to associative auditory cortex • In 2 UWS: no activation In 3 UWS: primary auditory cortex In 2 UWS: primary auditory cortex extending to associative auditory cortex (clinical improvement to MCS at three-month follow up)*	Di et al. (2007)
fMRI	1 UWS	No	Passive listening to own vs. other names	Medial prefrontal cortex bilaterally (also activation in L temporo-parietal and superior frontal cortices)	Staffen et al. (2006)
PET	1 MCS	Yes	Passive listening to own name	Precuneus and anterior cingulate/mesiofrontal cortex (also activation in bilateral angular gyri, R temporo-parietal junction, L dorsal prefrontal regions and Broca’s area, bilateral posterior superior temporal sulci and dorsal superior temporal gyri, encompassing Wernicke’s area)	Laureys et al. (2004)

*ACC, anterior cingulate cortex; cACC, caudal part of the anterior cingulate cortex; SMA, supplementary motor area*.

**Figure 1:**
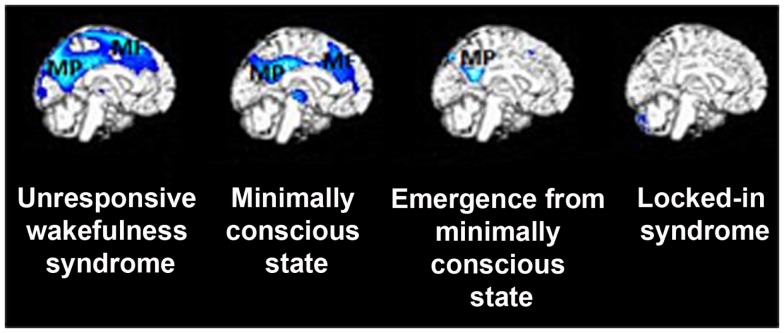
**Metabolic activity in medial precuneus (MP) and mesiofrontal (MF) cortex is severely impaired in patients with disorders of consciousness, such as in vegetative state/unresponsive wakefulness syndrome and minimally conscious state**. Of note is that patients who have emerged from the minimally conscious state (who yet experience confusion and amnesia syndromes) show metabolic dysfunction only in the posterior cingulate and adjacent retrosplenial cortex but not in the lateral frontoparietal network (see text). Finally, fully conscious yet severely paralyzed patients with locked-in syndrome do not show metabolic impairment in any of these areas, suggesting a critical involvement of midline regions in supporting self-related cognition (figure adapted fromThibaut et al., 2012).

Apart from activation studies utilizing self-referential stimuli, increasing attention has been paid to spontaneous brain activity and its significance to self-related cognition. During resting state, a set of brain areas encompassing precuneus, medial prefrontal cortex and bilateral temporo-parietal junctions have been shown to work by default, when subjects do not perform any task (Gusnard and Raichle, 2001). This default mode network (DMN) of areas in healthy controls has been related to internally oriented cognitive content, such as self-referential or social cognition, mind-wandering, and autobiographical memory recall (e.g., D’Argembeau et al., 2005; Mason et al., 2007; Buckner et al., 2008; Schilbach et al., 2008; Vanhaudenhuyse et al., 2011). Such intrinsic cerebral activity also seems to be implicated in consciousness processes. For example, in brain death, where all brainstem reflexes are lost parallel to continuous cessation of respiration, functional connectivity in the DMN is absent (Boly et al., 2009), or attributed merely to motion artifacts (Soddu et al., 2011). Coma patients show no identifiable fMRI DMN connectivity and in those patients where such connectivity can be detected may indicate subsequent recovery of consciousness (Norton et al., 2012). In patients with disorders of consciousness, such fMRI DMN connectivity is partially preserved yet severely disrupted, showing consciousness level-dependent decreases when moving from healthy controls to patients in MCS, VS/UWS, and coma (Vanhaudenhuyse et al., 2010). Interestingly, EEG studies have corroborated these findings: it has been shown that the strength of DMN EEG synchrony was smallest or even absent in patients in VS/UWS, intermediate in patients in MCS, and highest in healthy fully self-conscious subjects (Fingelkurts et al., 2012). Similarly, brain metabolism in these midline structures is severely disrupted in patients in VS/UWS and MCS compared to patients who have emerged from the MCS or are in a locked-in syndrome (Figure 1; Thibaut et al., 2012). It has been further proposed that deactivation of the DMN is supposed to reflect interruptions of introspective processes. Such investigation in patients showed that, compared to healthy controls, deactivation in medial regions of the DMN was absent in patients in VS/UWS and reduced in patients in MCS (Crone et al., 2011). Taken together, studies of spontaneous activity in patients suggest that changes in the DMN functional connectivity could suggest modified self-related conscious mentation. Indeed, it has been suggested that in normal waking conditions, resting state activity in the posterior cingulate, and frontal areas accounts for self-referential thoughts (Whitfield-Gabrieli et al., 2011; Fingelkurts et al., 2012). Therefore, it could be inferred that decreased connectivity in these midline regions of the DMN reflects, at least to certain degree, restricted abilities for self-referential processing in patients with disorders of consciousness.

## The Self as a Product of a Dynamic System Approach

Since the early studies of resting state, it has been suggested that the brain’s baseline activity can be organized in two brain networks showing anticorrelated activity to each other: an “intrinsic” and an “extrinsic” network (Fox et al., 2005; Fransson, 2005; Golland et al., 2007; Tian et al., 2007). The “intrinsic” network coincides with the DMN and is involved in the same cognitive processes as the DMN. The “extrinsic” system encompasses lateral frontoparietal areas resembling the brain activations during goal-directed behavior and it has been linked to cognitive processes of external sensory input, such as somatosensory (e.g., Boly et al., 2007), visual (e.g., Dehaene and Changeux, 2005), and auditory (e.g., Brunetti et al., 2008). Previous studies showed that these two systems are of a competing character in the sense that they can disturb or even interrupt each other (e.g., Tian et al., 2007). Such anticorrelated pattern is also illustrated in activation studies on motor performance (Fox et al., 2007), perceptual discrimination (Sapir et al., 2005), attentional lapses (Weissman et al., 2006), and somatosensory perception of stimuli close to somatosensory threshold (Boly et al., 2007).

We have recently proposed that these two systems may account for the phenomenological complexity of awareness. In particular, it is proposed that awareness, or the contents of consciousness, can be reduced to two components, namely the “external” awareness or everything we perceive through our senses (what we see, hear, feel, smell, and taste) and “internal” awareness or stimulus-independent thoughts (Demertzi et al., 2013). Interestingly, the switch between the external and internal milieu was found not only to characterize overt behavioral reports but also had a cerebral correlate (Vanhaudenhuyse et al., 2011). More particularly, it was shown that behavioral reports of internal awareness were linked to the activity of midline anterior cingulate/mesiofrontal areas as well as posterior cingulate/precuneal cortices. Conversely, subjective ratings for external awareness correlated with the activity of lateral fronto-parieto-temporal regions. These findings highlight that the anticorrelated pattern between the internal and external awareness system is of functional relevance to conscious cognition. Indeed, in an altered conscious state like hypnosis, where subjects report awareness alterations but remain fully responsive, hypnosis-related reductions in functional connectivity were shown in the external awareness system parallel to subjective ratings of increased sense of dissociation from the environment and reduced intensity of thoughts about external events (Demertzi et al., 2011). Similar reductions in external awareness systems have been also shown for non-responsive conditions, such as deep sleep and anesthesia (for a review, see Heine et al., 2012).

Analysis of metabolic activity obtained in VS/UWS patients compared to healthy controls or comparisons with recovery of awareness (i.e., within-subject), have highlighted the critical role of a widespread fronto-temporo-parietal associative cortical network (Thibaut et al., 2012). Recent PET data indicate that recovery of MCS patients seems to be accompanied by a right-lateralized recovery of the external awareness network whereas the presence of command following, defining the MCS plus (Bruno et al., 2011), classically parallels the recovery of the dominant left-lateralized language network (Bruno et al., 2012). Similar results have been observed in slow wave sleep and general anesthesia (for review, see Boveroux et al., 2008). Interestingly, these findings are also confirmed in transient dissociative states of unresponsive wakefulness, such as absence seizures, complex partial seizures, or sleepwalking – all characterized by preserved automatic reflex motor behavior in the absence of response to commands and showing transient impaired activity in these fronto-temporo-parietal associative areas (Laureys, 2005; Blumenfeld, 2012).

According to a suggested framework taking the external and internal awareness systems into account, two complementary states of system imbalance are possible, where one system can be in a hyperfunctional state, while the other is hypoactive. Extrinsic system hyperfunction is expected to lead to a state of total sensorimotor absorption or “lost self.” In contrast, intrinsic or default system hyperfunction is expected to lead to a state of complete detachment from the external world. A state where both extrinsic and intrinsic systems are hypofunctional is predicted to lead to markedly impaired consciousness as seen in disorders of consciousness (Soddu et al., 2009). A more recent proposal, adopting a similar system-level approach, points to the functional separation of the dorsal and ventral subcomponents of the posterior cingulate cortex (PCC): the ventral PCC appears to be highly integrated within the DMN, and is involved in internally directed cognition (e.g., memory retrieval and planning) whereas the dorsal PCC shows a highly complex pattern of connectivity, with prominent connections to the frontal lobes (Leech et al., 2012). According to the suggested model, differential regional activity can be explained by considering the arousal state, the milieu of attention (internal vs. external) and the breadth of attention (narrow vs. broad) (Leech and Sharp, 2013). The model proposes that through its interactions with the prefrontal cortex, the dorsal PCC is involved in controlling attentional focus. Hence, interactions of these PCC sub-regions with other intrinsic connectivity networks are then involved in shifting the balance of attention along an internal/external and broad/narrow dimension (Leech and Sharp, 2013).

Taken together these studies indicate that DMN and anticorrelated external awareness system activity underlies (at least partially) conscious ongoing mentation. It should be mentioned that fMRI anticorrelations were previously subject to debate in the literature. It has been argued, for instance, that fMRI functional anticorrelations are nothing more than noise in the signal due to regression of the brain’s global activity during data preprocessing (Anderson et al., 2011). Other data, however, suggest that the anticorrelations persist both with and without global signal regression, suggesting some underlying biological origins for this anticorrelated pattern (Fox et al., 2009; Chai et al., 2012). We would agree with the latter evidence which is supported by studies in unconscious conditions, such as anesthesia, sleep, and in unresponsive patients (Boly et al., 2009) where these anticorrelations generally reduce or even disappear, accounting for their functional contribution to conscious cognition.

## Conclusion

Neuroimaging activation and resting state studies indicate an indirect measure of self-related cognition encompassing midline and lateral frontoparietal areas. Furthermore, such studies have recently shown a much more complex, dynamic, and multifaceted architecture of brain functional connectivity in the emergence of consciousness than previously thought. Importantly for non-communicating patients suffering from disorders of consciousness, such neuroimaging measurements are of medical and ethical importance (Jox et al., 2012). What remains to be determined is the clinical translation of these technologies which will allow medical professionals and families to better comprehend these disorders, plan efficient medical management, and in a far reaching perspective, to acquire new opportunities to restore their brain functions.

## Conflict of Interest Statement

The authors declare that the research was conducted in the absence of any commercial or financial relationships that could be construed as a potential conflict of interest.
